# Digging in a 120 years-old lunch: What can we learn from collection specimens of extinct species?

**DOI:** 10.1371/journal.pone.0270032

**Published:** 2022-07-06

**Authors:** Catarina J. Pinho, Vicente Roca, Ana Perera, Amanda Sousa, Michèle Bruni, Aurélien Miralles, Raquel Vasconcelos

**Affiliations:** 1 Laboratório Associado da Universidade do Porto, CIBIO-InBIO, Centro de Investigação em Biodiversidade e Recursos Genéticos, Vairão, Portugal; 2 Faculdade de Ciências da Universidade do Porto, Departamento de Biologia, Porto, Portugal; 3 BIOPOLIS Program in Genomics, Biodiversity and Land Planning, CIBIO, Vairão, Portugal; 4 Facultat de Ciències Biològiques, Departament de Zoologia, Universitat de València, València, Spain; 5 Collections Scientifiques (Direction du Développement), Musée Océanographique de Monaco, Monaco, Monaco; 6 Institut de Systématique, Évolution, Biodiversité (ISYEB), Muséum National d’Histoire Naturelle, CNRS, Sorbonne Université, EPHE, Université des Antilles, Paris, France; State Museum of Natural History, GERMANY

## Abstract

Studying collection specimens is often the only way to unravel information about recent extinctions. These can reveal knowledge on threats and life traits related to extinction, and contribute, by extrapolation, to the conservation of extant species. However, high-throughput sequencing methods have rarely been applied to extinct species to reveal information on their ecology. Insular species are especially prone to extinction. We studied the gut contents of three specimens of the extinct giant skink *Chioninia coctei* of the Cabo Verde Islands using microscopy and DNA-metabarcoding. The presence of *Tachygonetria* adult nematodes suggests plants as important diet items. Our metabarcoding approach also identified plants and, additionally, invertebrates, supporting the hypothesis of *C*. *coctei*’s generalist diet. The absence of vertebrates in the digestive contents may reflect the decline of seabirds on the Desertas Islands that could have contributed to the debilitation of the giant skink, already depleted by persecution and severe droughts. Even with a small sample size, this study contributes to shedding light on the trophic roles of this enigmatic extinct species and emphasizes the need to develop holistic conservation plans for island threatened taxa. Additionally, it illustrates the potential of integrating up-to-date molecular methods with traditional approaches to studying collection specimens to help to solve ecological puzzles in other ecosystems.

## Introduction

Anthropogenic threats and climate change are driving more and more species to extinction faster than the discovery rate, leading to a global concern on how to halt the current biodiversity loss [1,2]. Island ecosystems present the highest rates of both unique biodiversity and species extinctions [3]. Even though the number of species is typically low compared to the mainland, the number of endemics is usually high [3]. Low gene flow among islands, together with lower predation pressures and more limited food resources, promote the occurrence of unusual biological characteristics, such as diet specialization [4–6], and gigantism [7–10]. However, these traits have been shown to make species more vulnerable to extinction [11–13].

Looking into the past and learning more about extinct species can unravel important information about the threats and traits related to their extinction and help to develop adequate conservation actions for closely related extant species [14–17]. One of the approaches to achieve this consists of taking advantage of the recent development in molecular biology in order to shed new light on ecology by studying historical collection specimens [18–20]. Several studies have used DNA from historic museum specimens to obtain important temporal evolutionary perspectives [21,22]. However, few have focused on extinct island species of reptiles [23]. Most recently extinct lizard species were island endemics, and frequently larger than extant ones [7,24]. Extinction processes have been associated with anthropogenic pressure, affecting even more habitat specialists inhabiting reduced areas [25]. Insular reptiles often exhibit peculiar feeding habits [4,26,27] such as herbivory [8,27,28], omnivory or cannibalism, thus, playing valuable roles in the ecosystem as seed dispersers, pollinators and even top predators in some cases [29]. Therefore, studying their diet, ecology, and behaviour can help to raise awareness of humans about the importance of reptiles to enhance the survival of other island threatened species.

**Fig 1:**
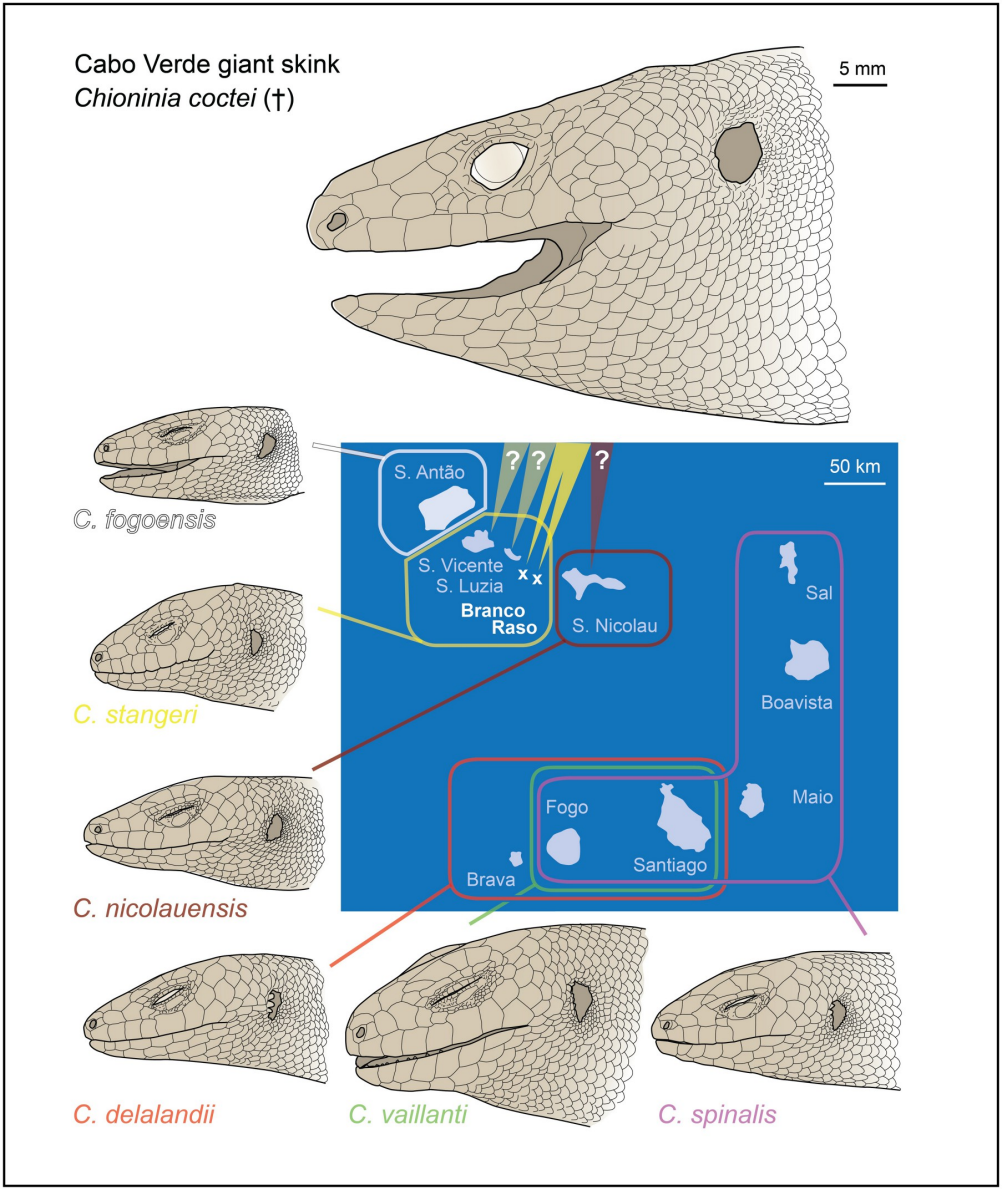
Extant and extinct (†) *Chioninia* species of Cabo Verde Islands. Distribution map of the seven different endemic *Chioninia* species, with illustrations showing their remarkable size variation.

The Cabo Verde Archipelago is nearly 500 km off the Atlantic African coast and comprises ten main islands and several islets (Fig 1). This biodiversity hotspot has a remarkable reptile diversity and all native taxa are endemics [30,31]. The *Chioninia* genus holds all the endemic skinks found in Cabo Verde, namely six extant species and the extinct Cabo Verde giant skink, also known as Cocteau’s skink, *Chioninia coctei* (Duméril & Bibron, 1839) [32], which present remarkable adaptive characteristics [33]. This emblematic species, which became extinct more than one century ago, was one of the largest skinks in the world. Its large dimensions (snout-vent length reaching up to 380 mm [34]), along with the five-cuspid teeth, unique dorsal and cephalic scales [32], and the diversified colouration pattern [35], made this species not only remarkably divergent from the six other species of *Chioninia* (Fig 1) but also from the ca. 1700 known species of Scincidae [32,34–37].

The Cabo Verde giant skink is classified as Extinct since the second half of the 20th century by the International Union for Conservation of Nature (IUCN) Red List of Threatened Species [38] and in the First Red List of Cabo Verde Reptiles [39]. It was last seen in its natural habitat in 1912 [40], despite repeated subsequent searches [31], plus a last unsuccessful mission performed by the authors on Branco in 2017. During the 19th century, its presence was unambiguously demonstrated on the two islets of Branco and Raso [30,34], and subfossil reports and museum specimens confirm the original distribution range of *C*. *coctei* on the islands of Santa Luzia and São Vicente [41,42]. Even though in 2005 a mandible of a juvenile was recovered in cat scats on Santa Luzia [43], a 2006 extensive survey on the island failed to find indications of the living presence of this species [31,32]. It might have also been present in the island of São Nicolau as stated by local fishermen, though so far, no solid evidence was found [40,44]. The probable causes of Cocteau’s skink extinction seem to rely on a combination of human and natural causes. The introduction of mammals in the archipelago may have had a significant impact on the number of specimens, as well as repeated long drought periods [45]. Due to its uncommonly large size, this species was actively hunted by fishermen and prisoners deported in 1833 to the uninhabited Branco Islet, either as a food source [40], for their supposed medical properties, or to use their skins [34,46]. Furthermore, this ‘giant’ skink attracted the attention of natural history dealers, which led to the collection of at least 86 specimens (but very likely more) for museums and private collections (see S1 Table and S1 Fig) [41,45]. Based on the literature and museum data at least 63 specimens were catalogued during 1880–1930 (S1 Table and S1 Fig).

The knowledge on the ecology of *C*. *coctei* is scarce and mainly relies on empirical and scattered historical observations. Most authors, based on direct observations and interviews of locals, referred to its commensalism with seabirds, such as the Cabo Verde shearwater *Calonectris edwardsii* (Oustalet, 1883), where lizards occupied rocky burrows of nesting sites and consumed the leftovers of regurgitations that adult birds spill over while feeding their chicks [40,47,48]. Also, Alexander [47] reported that skinks had been pulled out from the holes of white-faced storm petrels *Pelagodroma marina* (Latham, 1790). Nocturnal or crepuscular according to Vaillant [49], this species would prey on seabirds’ eggs and insects. Its particular teeth morphology has been regarded by several authors as evidence of an adaptive shift toward a herbivorous or omnivorous diet [50,51]. It was also reported that animals kept in captivity survived feeding exclusively on plants [50,52]. Despite these invaluable past reports representing the few direct testimonies about the species, they are contradictory, and more data would be necessary to unravel the diet and other ecological features of this species.

Even though no more living individuals can be found, this charismatic species can still be studied through natural history collection vouchers. Their digestive tract contents represent the only remaining source of evidence to determine the species diet. DNA based technologies such as next-generation sequencing (NGS) metabarcoding have demonstrated their potential to recover valuable information from degraded collection specimens [53–55]. In this study, we take advantage of those technologies to shed light on the enigmatic ecology of this extinct species and to explore possible reasons that may have contributed to its extinction. Particularly, we aimed to learn more about the health status and diet of the last three known specimens of giant skink collected in 1901 on Branco Islet by the Princess Alice II expedition and which, according to our knowledge, have never been examined by scientists so far.

## Methods

### Type locality

Branco Islet, located between Santa Luzia Island and Raso Islet, is part of the Desertas Islands in the Cabo Verde Archipelago. This central island group is characterized by a dry tropical Sahelian climate, with very arid plane lowlands and medium elevation areas [56]. Branco, which emerged around 6 million years ago, is only 3 km^2^ [57], and it is dominated by mountainous and medium-elevation arid areas, reaching its highest point in Tope Berta (353 m) [58]. This islet is of difficult access due to the roughness of the sea, lack of safe natural ports, steepness (there is just a small flat area of about 400 × 200 m), and high exposure to the trade winds in most of the north coast. The Desertas Islands present an annual rainfall ranging between 122–186 mm (mean = 148.5 ± 15.9 mm) [58].

### Examination of specimens

In 2017, during a visit to the ‘Musée océanographique de Monaco’ (MOM), at the ‘Institut Océanographique’, Fondation Albert I^er^, Prince de Monaco, five vouchers of *Chioninia coctei* (VS0000067_A–E), never referred to in the scientific literature before were discovered and studied. The specimens were collected on July 22^nd^ and 23^rd^ of 1901 by Prince Albert I on Branco Islet during the Expeditions of Princess Alice II [59]. The five specimens were photographed dorsally and ventrally, measured from the tip of the snout to the posterior edge of the cloaca (SVL), and sexed by examining the presence/ absence of hemipenes and in the case of dissected individuals, their internal reproductive structures. The photos of the specimens were deposited in Morphobank (Table 1). We were allowed to dissect three of them. The digestive tracts of these specimens (hereafter individuals A, B and C) were opened with a scalpel and examined. A considerable number of parasites were observed while collecting the tracts. The digestive contents were collected and preserved in 70% pure ethanol and stored at -20ºC for further analyses. At the end of this study, voucher A (UCV2017/0001) was donated by MOM to the collections of the Technical University of the Atlantic, São Vicente, the future Natural History Museum of Cabo Verde.

**Table 1:** Study specimens.

Code	SVL (mm)	Sex	Diet	Nr of helminths	Morphobank code
VS0000067_A*	240	F	Yes	236	M687941; M687942
VS0000067_B	267	M	Yes	12500 aprox.	M687944; M687945
VS0000067_C	228	M	Yes	0	M687946; M687947
VS0000067_D	237	?	No	-	M687948; M687949
VS0000067_E	188	M	No	-	M687950; M687951

Details of the *Chioninia coctei* vouchers rediscovered in ‘Institut Océanographique’ Fondation Albert Ier, Prince de Monaco. All specimens were collected on Branco Islet in 1901.

* This voucher was recently donated to the Natural History Museum of Cabo Verde (UCV2017/0001).

### Parasite analysis

Adult helminths were removed from the digestive contents and morphologically examined with a binocular magnifying glass. The helminths were photographed, counted, and taxonomically identified based on the following variables: oesophagus length, the structure of the apical end, the structure of the caudal end of the males—including length and shape of the spicule, existence and shape of the gubernaculum, and arrangement of the caudal papillae [60–62]. Then, helminths were preserved in 70% ethanol.

In addition, the DNA of the helminths was extracted using QIAamp DNA Micro Kit (Qiagen, Crawley, UK), following the manufacturer’s instructions. The DNA amplification was carried out for the nuclear 28S rDNA, including the D2 region, using the primers 28SF0001 and 28SR0990 (~850 bps) [63]. The PCR was performed in a total of 10 μL reaction volumes containing: 2 μL of QIAGEN Multiplex PCR Master Mix (Qiagen, Crawley, UK), 0.5 μL of each 10 μM primer, 3 μL of ultra-pure water, and 4 μL of DNA extract. Cycling conditions used an initial denaturing at 95ºC for 12 min, followed by 35 cycles of denaturing at 95ºC for 15s, annealing at 50ºC for 1 min and extension at 72ºC for 1 min, with a final extension at 72ºC for 7 min. Post-PCR steps (PCR clean-up, cycle sequencing, cycle sequencing clean-up) followed standard procedures for degraded DNA [64]. PCR products were in a first attempt Sanger sequenced and in a second attempt sequenced on Illumina’s MiSeq platform [64].

### Metabarcoding diet analysis

The stomach contents were air-dried completely to reduce the volume and concentrate the DNA. DNA extraction was performed using the whole homogenised stomach contents, in several total volumes of 50 μL, using the Stool DNA Isolation Kit (Norgen Biotek Corp.Canada) and following the manufacturer’s instructions.

Three different DNA markers were selected to identify the diet items present in the digestive contents. For plants, the g/h primers that target the short P6-loop of chloroplast trnL (UAA) intron (~10–143 bps) were used [65]. The primers IN16STK-1F/ IN16STK-1R, targeting the mitochondrial 16S rRNA gene (~110 bps) [66] and 12sv5F/ 12Ssv5R targeting the V5-loop fragment of the mitochondrial 12S gene (~73–110 bps) [67] were used to amplify invertebrates and vertebrates [67], respectively. All primers were modified to contain Illumina adaptors and individual barcodes to allow individual identification for the three genetic loci. The regions were amplified by PCR cycles in a total volume of 25 μL, following specific conditions according to the taxonomic group (following [68]). Library preparation was carried out following Illumina MiSeq protocol 16S Metagenomic Sequencing Library Preparation [69]. The final library was run on a MiSeq sequencer (Illumina, San Diego, CA, USA) using a 2 × 150 bp MiSeq Reagent Kit (Illumina, San Diego, CA, USA) for a projected average of 12,000 paired-end reads per sample.

The software package OBITools (http://metabarcoding.org/obitools) was used for sequence processing. After filtering, the final haplotypes were blasted against known reference sequences in the GenBank database (https://www.ncbi.nlm.nih.gov/genbank/) and our Molecular Operational Taxonomic Units (MOTU) reference database [68]. Sequences with less than 90% of similarity were classified only to the class level, whereas the ones with values between 90% to 95% were classified to the family level. In addition, sequences with similarity values beyond 95% were classified to the genus or species level. If a haplotype was similar to more than one species or genus, only species or genera recognized to occur on Branco Islet, or the surrounding islands of the archipelago were considered [70]. Haplotypes identified as contaminations (e.g., human DNA) were removed.

## Results

### Examination of specimens

Vouchers had an average SVL of 232 mm (n = 5, Table 1). Voucher A was identified as a female; voucher D was not possible to sex, and the remaining specimens were identified as males (Table 1). Individuals A, B and E appeared to be unusually skinny and/or dehydrated (see photos on Morphobank).

### Parasite analysis

**Fig 2:**
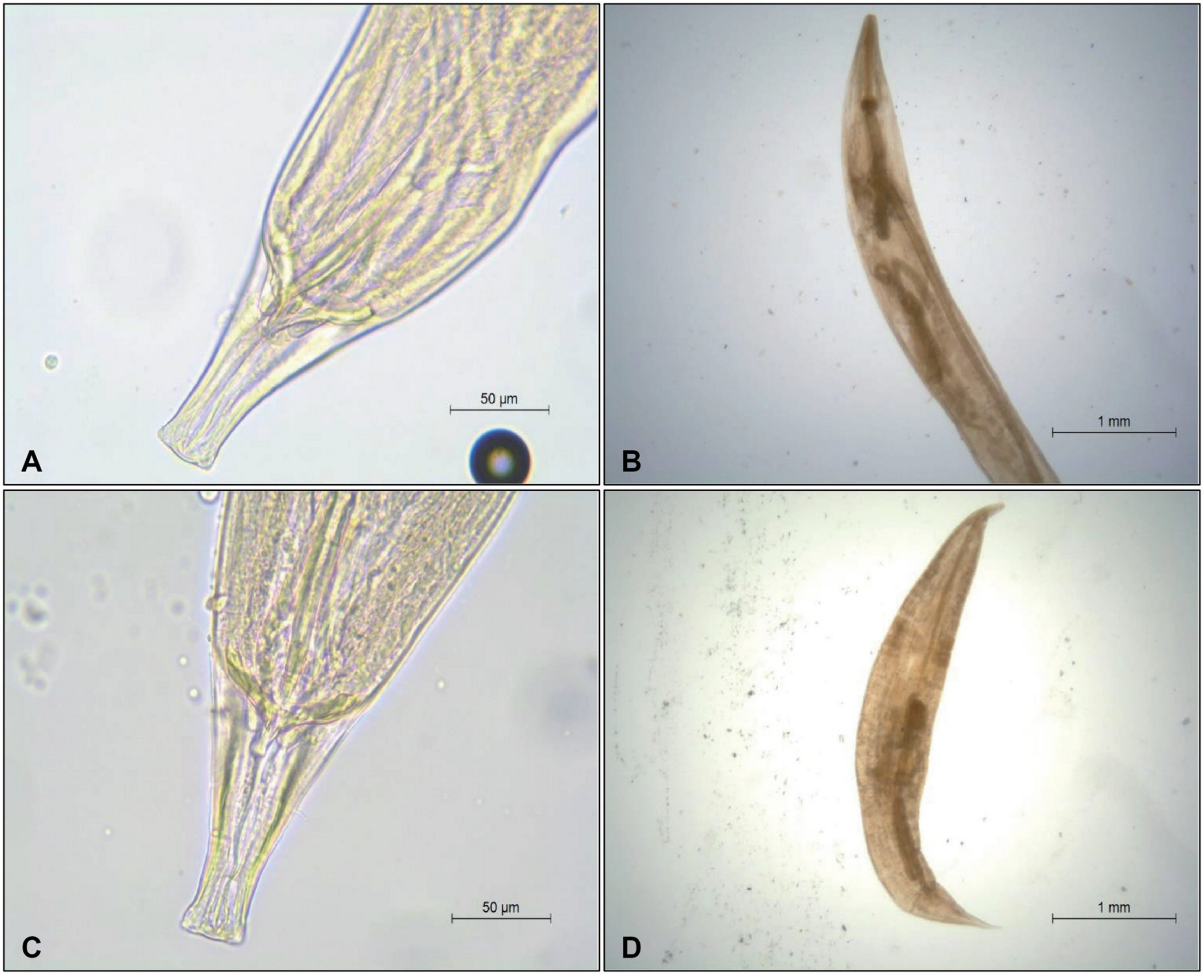
Ventral views of *Tachigonetria* nematodes identified from *Chioninia coctei*’s digestive contents (photos by V. Roca). A) *T*. *longicollis*, male caudal end (only *T*. *l*. *longicollis* is represented as differences between subspecies are difficult to depict); B) *T*. *numidica*, female; C) *T*. *macrolaimus*, male caudal end; D) *T*. *conica*, female.

The analysis of the intestine contents revealed the presence of nematode parasites in two of the three specimens analysed, all of them belonging to the family Pharyngodonidae (Table 1). All sampled nematodes were identified morphologically to species-level based on the above-mentioned morphological variables (Fig 2A–2D). Five different taxa were identified, all belonging to the same genus *Tachygonetria* Wedl, 1862: *T*. *longicollis longicollis* (Schneider, 1866), *T*. *longicollis setosa* (Petter, 1966), *T*. *macrolaimus* (Linstow, 1899), *T*. *numidica* Seurat, 1918, and *T*. *conica* (Drasche, 1884). In specimen A, we isolated a total of 236 nematodes, all females of the first three taxa. In voucher B, it was possible to observe a compact mass of plant matter to which a large number of nematodes were attached. In this voucher, thousands of nematodes were found, both males and females, with an estimated amount of approximately 12.500 individuals belonging to the five species mentioned above (Table 1). On the intestinal contents of voucher C, no nematodes were observed. Despite several attempts, sequencing failed for the DNA samples of the nematodes, probably due to the highly degraded state of the samples.

### Metabarcoding diet analysis

**Fig 3:**
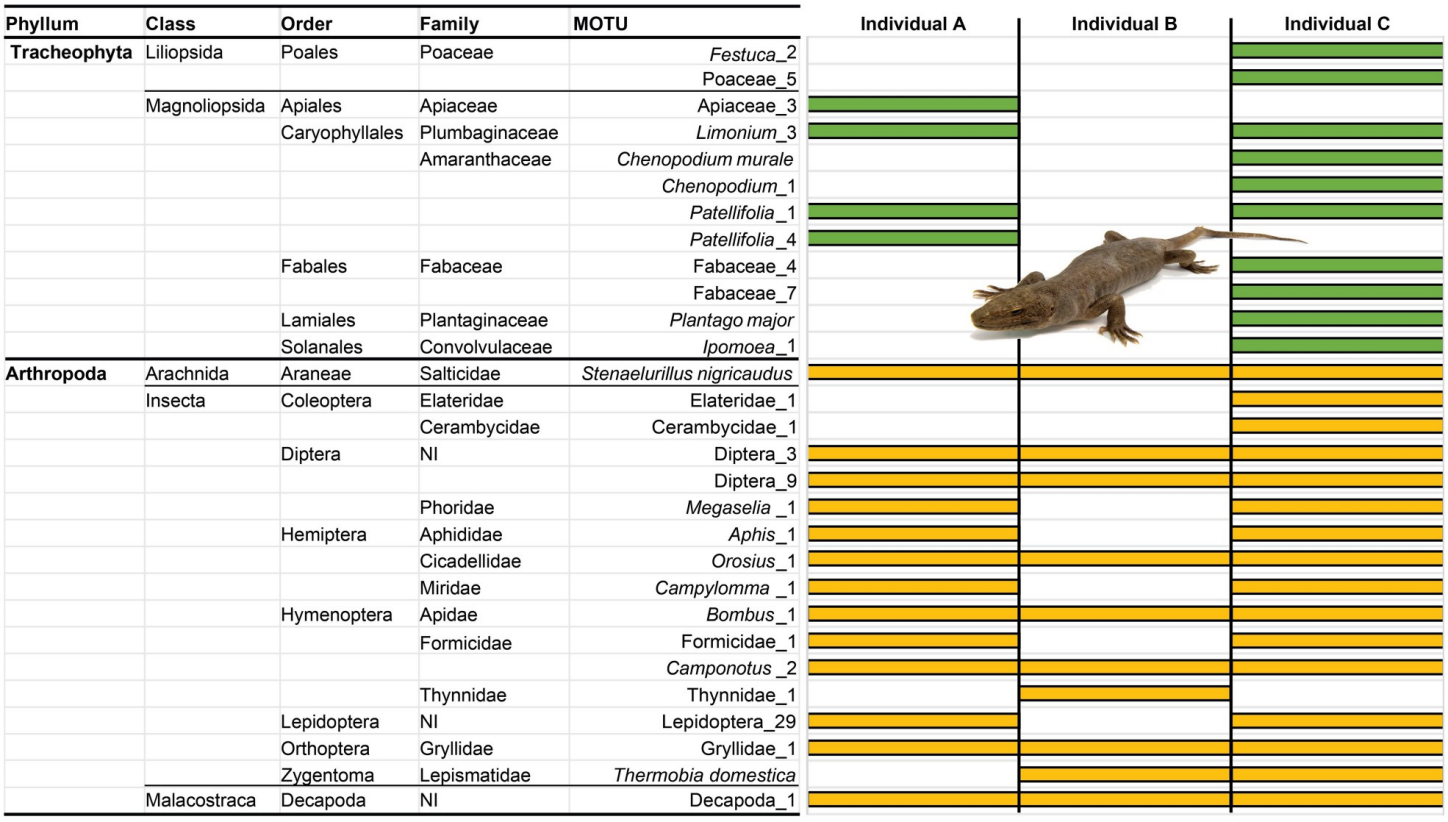
Diet items detected in the stomach contents of the *Chioninia coctei* vouchers (photo by M. Dagnino). Items are represented until the higher taxonomic identification possible to obtain. Plant and invertebrate MOTUs are represented in green and yellow, respectively. NI describes non-identified families.

The overall diet composition of the three studied specimens, obtained with metabarcoding, is depicted in Fig 3. After bioinformatic filtering, the average coverage obtained was about 11200 sequence reads per sample (see S2 Table). We identified a total of 29 diet items, 12 corresponding to plants—comprising six orders and seven families—and 17 to arthropods—comprising nine orders and 12 families. The haplotype sequences of the 29 MOTUs are available in the S2 Table. The analysis identified plant items in two of the three samples and invertebrates in all three samples. Sample A presented four MOTUs of plants and 13 of invertebrates, sample B 10 MOTUs of invertebrates, and sample C 10 of plants and 16 of invertebrates. No vertebrate DNA sequences were detected. Plumbaginaceae (e.g.: *Limonium*) and Amaranthaceae (e.g.: *Patellifolia*) plant families had the highest frequency of occurrence for plants. Five arthropod families were present in all samples (Salticidae, Cicadellidae, Apidae, Formicidae, Gryllidae), having the highest overall frequency of occurrence, along with non-identified Diptera and Decapoda (Fig 3).

## Discussion

Even though our results only represent a very small part of the last extant population of the Cabo Verde giant skink and a snapshot of its diet, as we studied only the last meal ingested by three specimens, we provide valuable data regarding the conditions and dietary habits of this emblematic extinct species. Considering this is an extinct and rare species, with roughly 80 specimens in worldwide museums, of which only about 30 specimens are putatively available, getting information from three vouchers, corresponding to 10% of all vouchers (see S1 Table and S1 Fig), represents the first opportunity to describe the diet of this species and provide unique clues on the ecology of this enigmatic species.

### Parasites and herbivory hypothesis

The report from the Princess Alice II expeditions described the captured individuals as ‘miserable’ looking [52]. This gives us clues on the health status of the last *C*. *coctei* individuals of a population on the brink of extinction. This observation may be related to the extremely high parasitic load of one of the vouchers that we found. Naturally, a sense of balance between parasites and reptile hosts prevents the progress of diseases, however, if exposed to environmental stressors this equilibrium can be broken [71,72]. In this latter case, the health of the host can be directly or indirectly affected by helminths [73]. Host social behaviour can be modified by parasites promoting aggressive actions, reducing mobility which can reduce the possibility of finding a partner or food, reducing reproduction investment by females [74] and ultimately survival rates [75,76]. However, the other two vouchers presented parasite loads similar to the ones observed in other large insular lizards in the nearby Canary Islands [77,78], positing to the existence of other main drivers affecting the health of the individuals.

Several studies showed an association between the nematode fauna and the type of diet of the reptile hosts [77,79,80]. The helminths from the Pharyngodonidae family have two evolutionary lineages, comprising different genera, commonly associated with different diets, carnivorous saurians or herbivorous iguanids/ tortoises [81]. The genus *Tachygonetria* identified in our samples corresponds to the lineage that usually infects herbivorous reptiles. Their higher incidences are typically found in tortoises and lizards with omnivorous or herbivorous diets [82–84]. The finding of *Tachygonetria* in our study suggests an, at least partial, herbivorous diet of *C*. *coctei*. This lineage could have infected this Cabo Verde reptile through the endemic and extinct Cabo Verdean tortoise *Geochelone atlantica* López-Jurado *et al*. 1998, as they could easily disperse between islands carrying nematodes with them, or by host switching with another infected sympatric herbivorous host [85], such as *Chioninia stangeri* (Gray, 1845) lizards. Unfortunately, our unsuccessful attempts to sequence these nematodes prevented us from disentangling the evolutionary history of the host-parasite interactions and the origin of these nematodes. Since Pharyngodonidae has a typical monoxenous life cycle, i.e., infecting a single host, it has been proposed [80] that the infection of herbivorous reptiles is favoured as they may have greater chances of inadvertently ingesting eggs dropped in plants through faecal pellets of infected hosts [80]. Besides, an intensification of plant matter consumption offers an ideal environment for the development of more rich and diverse helminth communities [80,81,83].

Insular reptile populations can reach higher densities as terrestrial predators are generally scarce [86]. In the drier periods, where food resources are scarcer, plant items may play an important role in their diets to balance the lower availability of arthropod prey [87], exactly when our vouchers were collected. At the same time, very small distribution ranges favour the transmission of nematodes among individuals [88,89]. Large body and gut sizes favour the recruitment of nematodes and are usually associated with higher consumption of plant matter [90]. Both characteristics were recorded in the studied *C*. *coctei* specimens. However, the low caloric intake provided by plants and the need for longer digestions makes the individuals more susceptible to introduced predators and humans. Indeed, several old records describe how easily these animals were caught by hand [34,40,52,91].

### Omnivory hypothesis

Our molecular results also support the adaptation towards the consumption of plant matter by *C*. *coctei*. Plant consumption was identified in two out of three analysed samples, and even though we could not amplify plants in sample B, it was possible to observe traces of plants (e.g., green, and fibrous mass) when we examined the digestive content under the binocular magnifying glass. So, all studied individuals consumed plants. The morphology of the conic teeth with five cuspids [34,50], typical of herbivores [44], and empirical studies describing that some captured specimens survived several years feeding only on plants, gave origin to the assumption that the species was exclusively herbivorous [51]. However, the diet of *C*. *coctei* was mostly referred to in the literature as a generalist [44,48,49]. Our results support this hypothesis since we did find arthropods in all the three digestive contents analysed. And, in fact, some invertebrates were present in all samples (e.g., Decapoda) and had a higher diversity of MOTUs than plants. Skinks could be directly feeding on Decapoda, as observed in other island reptile species, such as *Cryptoblepharus cognatus* (Boettger, 1881) in Madagascar [92]. Alternatively, the secondary consumption of marine organisms is expected considering the commensal link of the giant skink with seabirds [93]. In fact, several reptiles adapted to oceanic islands feed on seabird eggs or their regurgitations [44,68,94,95].

No traces of vertebrates were detected in the gut content of the three sampled specimens, precluding to confirm several historical reports mentioning predation of this skink on seabirds (e.g. [48]). These reports mentioned a commensal and predatory link with endemic seabirds such as the Cabo Verde shearwater *Calonectris edwardsii* [40,47,48], especially after bird nesting season [44]. The lack of bird sequences in digestive contents could have been explained if the samples were collected outside the birds breeding season, but this was not the case. Although the breeding season of Boyd’s shearwaters *Puffinus boydi* Mathews, 1912, white-faced storm petrels *Pelagodroma marina* and Cabo Verde storm petrels *Oceanodroma jabejabe* (Bocage, 1875) extend only until June, the more abundant Cabo Verde shearwaters usually breed from June to November [96]. The latter one, in particular, was severely persecuted for food by fishermen for centuries [97]. Besides, in 1833 prisoners were deported to the deserted Branco Islet [40], and without any available resources, along with the skinks themselves, the chicks and adult seabirds could have been a valuable food source. That led to a severe decline of the seabird populations on Branco, and their almost disappearance from Santa Luzia, where the skink first disappeared [96]. Even though the islet populations were safe from introduced mammals, the seabirds decline through persecution may have reduced the access by skinks to this highly energetic food resource, while contributing to their fitness debilitation, especially in drought periods [87]. Our study specimens were collected after several decades of overexploitation of these resources, which could explain our results. An alternative explanation for the absence of vertebrates could be due to the small sample size and technical limitations, such as the lack of power of the 12S primers to amplify the highly degraded DNA of our samples. However, these markers have been effectively used to amplify museum samples [98] and were used in a variety of dietary studies with successful results [68,99]. Moreover, the other markers worked very well on these samples despite being more than 100 years old and possibly poorly preserved for DNA amplification.

To disentangle the several hypotheses related to the lack of seabird sequences we could try to get permission to check the digestive contents of more specimens of the beginning and mid-19th century, even though the chances of getting quality DNA are very low [53]. Several specimens of *C*. *coctei* were recently rediscovered in other institutions, such as the ones in the Natural History Museum of the University of Porto and Passos Manuel Lyceum in Lisbon [100], and are waiting to be studied using other techniques (e.g. osteology, isotopic analysis) that could shed some light on this [45]. We could also check the diet of the co-occurring reptile species and indeed the geckos feed on seabirds and their regurgitations [68], which suggests that herbivory is a common strategy to survive in such harsh and limited resourced islet [44]. Unfortunately, no diet studies were conducted for the extant smaller *Chioninia* species, even though anecdotal observations reported they prey on insects and plants confirming an omnivorous diet [30].

### Alternative hypotheses for extinction drivers

In addition to the severe decline of seabirds, other potential factors may have contributed to *C*. *coctei* extinction. Droughts are a centuries-old problem in the archipelago and the 18^th^ and 19^th^ centuries were noticeable by long-term droughts that lead to severe famines and epidemics across the islands [101]. These also represented serious threats to biodiversity on islands, for instance, populations of *Alauda razae* (Alexander, 1898) fell to extremely low levels during these periods [102]. Consequently, long periods of droughts might have reduced drastically vegetation and arthropod availability on the islands. This skink presumably had a very low fecundity rate [45]. It is hypothesized that the average age at which sexual maturity was reached was five years for males and six years for females [45]. And, as an oviparous species [35,103], females normally laid two eggs per clutch [45]. All these factors that lead to the intensification of hunting of the skinks and seabirds [45], namely the introduction of mammal predators, such as cats and rodents, and the over-exploitation by natural history dealers, may have led to the extinction of the Cabo Verde giant skink [41,45]. All these extinction drivers were especially severe at the end of the 19th century, to the point that researchers feared its extinction at that time [87]. The example of this remarkable extinct species proves that species are vastly interdependent, especially on islands, as they co-evolved in disharmonic and isolated systems. Since the number of insular species is reduced, the trophic links among species are fewer and stronger, leading to a more fragile balance [104]. This is especially evident in the smaller, most isolated, low-elevation islands [104,105] like the Desertas. Consequently, the local disappearance or reduction of even one element of that network, such as shearwater, might have led to the collapse of this delicate equilibrium, especially at the top of the ecological chain [106], such was probably the case of *C*. *coctei*. For this reason, it is crucial to design conservation plans that focus on protecting the ecological processes and not individual species [107]. In this way, it will be possible to ensure that processes such as pollination, seed dispersal and predation continue their viability in island ecosystems that are more vulnerable to disruptions [29]. However, the development of holistic conservation plans relies on studies of the diet and trophic roles of endemic species that are generally lacking [94]. This is striking for reptiles and with our work, we hope to contribute to efficiently diminishing this shortfall by integrating classical morphological with cutting-edge genetic technologies (e.g. [99]). This study can foster conservation measures for the six extant *Chioninia* species, two of which are classified as Endangered in the IUCN Red List of Threatened Species [31].

### Genetics leveraging museum collections

Previous studies using DNA sequencing of ancient coprolites, rumen and gut microbiomes already provided valuable information on the diet of New Zealand’s Upland Moa *Megalapteryx didinus* (Owen, 1883), Yakutian bison *Bison priscus* (Bojanus 1827), woolly mammoth *Mammuthus primigenius* (Blumenbach 1799) and woolly rhinoceros *Coelodonta antiquitatis* (Blumenbach, 1799), highlighting the informativeness of molecular approaches to unravel species extinction causes [108,109]. Likewise, molecular studies of sub-fossil remains and collection vouchers have been important to reconstruct the phylogeography and demographic history of the Tasmanian tiger *Thylacinus cynocephalus* (Harris, 1808) presently extinct [110]. However, genetic methods of high-throughput sequencing, such as metabarcoding, have rarely been applied to extinct species to reveal information on habitat preferences, behaviour, and ecology [19,109].

Our study illustrates once more the potential of new molecular tools, even when applied to old and degraded digestive contents. It also emphasizes the value of collection specimens to obtain reliable data on the ecology of extinct species and to provide some clues about their extinction drivers. Disclosing the existence of these overlooked vouchers will allow further studies to be performed and to solve other pieces of the ecological puzzle of Cabo Verde and other understudied ecosystems.

## Supporting information

S1 Fig Number of *C*. *coctei* specimens catalogued in museums per 50-year period. Unknown clusters group all vouchers with unavailable cataloguing dates (check S1 Table for details).
(TIF)

S1 Table Voucher data published in the literature or available on candidate museum websites. Information regarding the voucher code, number (Nr), cataloguing date (Year), origin, museum name and location, the name of the collector/donor, and some notes are given. Listing according to ascending cataloguing date.
(PDF)

S2 Table Details on the diet items identified in the stomach contents of the *Chioninia coctei* vouchers. Taxonomic identifications, frequency of occurrence (FO), haplotype sequences and the respective number of reads of the diet items. The final ID of MOTUs corresponds to the highest taxonomical classification possible.
(PDF)

S1 Appendix Abstract in Portuguese. (PDF)
