# Supplementary figures and images for: Digging in a 120 years-old lunch: What can we learn from collection specimens of extinct species?
Source: PLoS One. 2022 Jul 6;17(7):e0270032. doi: 10.1371/journal.pone.0270032 (PMC9258829; doi:10.1371/journal.pone.0270032)

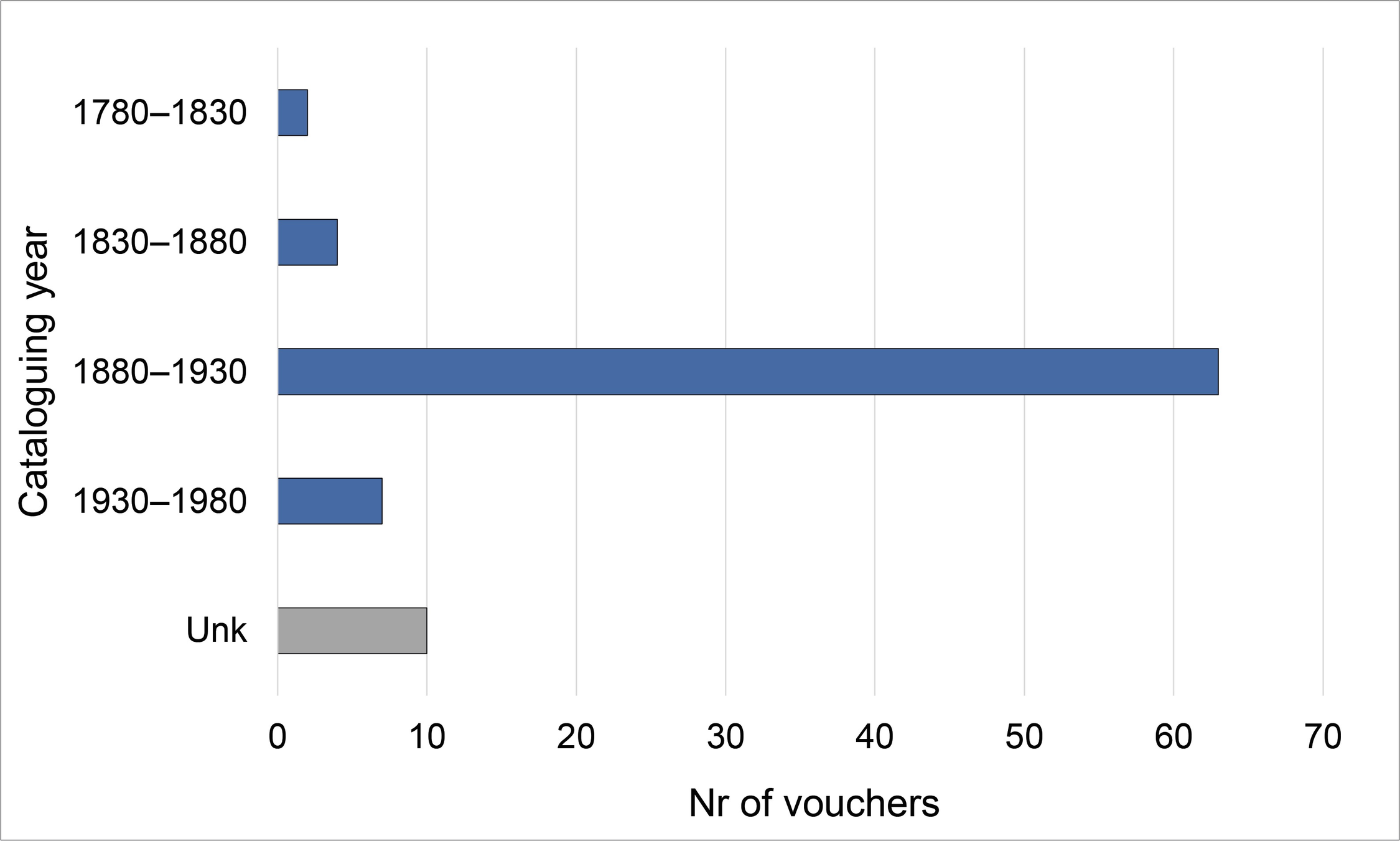

Supplement: S1 Fig — Unknown clusters group all vouchers with unavailable cataloguing dates (check S1 Table for details). (TIF) [file pone.0270032.s001.tif]
