# Supplementary material for: Digging in a 120 years-old lunch: What can we learn from collection specimens of extinct species?
Source: PLoS One. 2022 Jul 6;17(7):e0270032. doi: 10.1371/journal.pone.0270032 (PMC9258829; doi:10.1371/journal.pone.0270032)
Supplement: S1 Table — Information regarding the voucher code, number (Nr), cataloguing date (Year), origin, museum name and location, the name of the collector/donor, and some notes are given. Listing according to ascending cataloguing date. (PDF) [file pone.0270032.s002.pdf]

Voucher code	Nr	Year	Origin	Museum name	Location	Collector/donor	Notes
1891.9.24.1-2	2	1809	Cabo Verde	Natural History Museum	London, UK	-	Part taken from Lisbon in 1809
MNHN-RA-0.8299	1	1839	Branco	Muséum national d'Histoire Naturelle	Paris, France	João da Silva Feijó	Skin; part taken from Lisbon in 1809
RMNH.RENA.17057	1	1839	Branco	Naturalis Biodiversity Center	Leiden, Netherlands	João da Silva Feijó	Part taken from Lisbon in 1809
UP-MHNFCP - 017374	1	1874	Raso	Natural History Museum and Sciene	Porto, Portugal	D. Hopffer	
1877.9.26.1	1	1877	Cabo Verde	Natural History Museum	London, UK	-	
MNHN-ZA-AC-1891-1134	1	1881	Cabo Verde	Muséum national d'Histoire Naturelle	Paris, France	-	Skeletons
MNHN-RA-1884.148; MNHN-RA-1884.227, 228, 229, 231, 233, 236, 237; MNHN-RA-1987.941	9	1883	Branco	Muséum national d'Histoire Naturelle	Paris, France	Expédition scientifique du Navire 'Talisman'	
MSNG 8769.1-2	2	1891	Branco	Musei di Storia Naturale di Genova	Genova, Italy	D. Schiavetti	
-	2	1891	Branco	Museo delle Scienze	Trento, Italy	Mario G.Peracca	
MZUT R1981.1-26	26	1891	Branco	Musei di Zoologia e di Anatomia Comparata	Turin, Italy	Mario G.Peracca	26 adults, 6 eggs (MZUT R1981.27-32)
MZST Rl-6	6	1891	Branco	Museo Zoologico "G. Scarpa" di Treviso	Treviso, Italy	Mario G.Peracca	
NMW 10721 3/112/7	1	1892	Santa Luzia	Naturhistorisches Museum Wien	Vienna, Austria	Dr. Brillant	
MSNG 28891.1-2; 34516	3	1898	Raso	Musei di Storia Naturale di Genova	Genova, Italy	Leonardo Fea	
MZUF 176	1	1898	São Vicente	Museo Zoologico La Specola	Florence, Italy	G. Cecconi	
VS0000067_A	1	1901	Branco	Universidade Técnica do Atlântico	Cabo Verde	Prince Albert I	
VS0000067_B-E	4	1901	Branco	Musée Océanographique de Monaco	Monaco	Prince Albert I	
MNHN-RA-1906.295	1	1906	-	Muséum national d'Histoire Naturelle	Paris, France	Mario G.Peracca	
MNHN-ZA-AC-1906-458	1	1906	-	Muséum national d'Histoire Naturelle	Paris, France	Mario G.Peracca	Skeletons
NMW 885	1	1906	Cabo Verde	Naturhistorisches Museum wien	Vienna, Austria	Steindachner	
MNHN-ZA-AC-1907-255; MNHN-ZA-AC-1907-344	2	1907	Cabo Verde	Muséum national d'Histoire Naturelle	Paris, France	-	Skeletons
USNM 58495	1	1910	Cabo Verde	National Museum of Natural History	Washington, USA	-	https://www.gbif.org/pt/species/8412194
NMP-P6V 32744	1	1925	Cabo Verde	National Museum of Prague	Prague, Czech Republic	R. M. Boušek	Partially damaged adult
MNHN-ZA-AC-1941-227	1	1941	Cabo Verde	Muséum national d'Histoire Naturelle	Paris, France	-	Skeletons
MNHN-ZA-AC-1943-133	1	1943	Cabo Verde	Muséum national d'Histoire Naturelle	Paris, France	-	Skeletons
1960.1.8.3-4	2	1960	Cabo Verde	Natural History Museum	London, UK	-	
AMNH 7116	1	-	Cabo Verde	American Museum of Natural History	New York, USA	-	Hutchinson (1989) - skeleton
R.10491	1	-	Cabo Verde	Cambridge University Museum of Zoology	Cambridge, USA	-	https://www.gbif.org/pt/species/8412193
MSNG 43132	1	-	Branco	Musei di Storia Naturale di Genova	Genova, Italy	-	
-	1	-	Cabo Verde	Museum für Naturkunde	Berlin, Germany	-	Ceriaco 2015 - anonymus sine anno
-	1	-	Cabo Verde	Passos Manuel Secondary School	Lisbon, Portugal	-	
ZMS 1/1978; ZMS 1/1979; ZMS 1/1987	3	-	Cabo Verde	Zoologische Staatssammlung München	Munich, Germany	-	Schleich 1979/1982
R-21886	1	-	Cabo Verde	Museum of Comparative Zoology	Cambridge, USA	-	https://www.gbif.org/pt/species/8412194
RBINS 2325	1	1978-87	Cabo Verde	Royal Belgian Institute of natural Sciences	Brussels, Belgium	-	https://www.gbif.org/pt/species/8412194
