# Supplementary material for: Digging in a 120 years-old lunch: What can we learn from collection specimens of extinct species?
Source: PLoS One. 2022 Jul 6;17(7):e0270032. doi: 10.1371/journal.pone.0270032 (PMC9258829; doi:10.1371/journal.pone.0270032)
Supplement: S2 Table — Taxonomic identifications, frequency of occurrence (FO), haplotype sequences and the respective number of reads of the diet items. The final ID of MOTUs corresponds to the highest taxonomical classification possible. (PDF) [file pone.0270032.s003.pdf]

Phyllum	Class	Order	Family	Final_ID	FO	Haplotype sequence	Reads
Tracheophyta	Liliopsida	Poales	Poaceae	Festuca_2	0.33	atccgtgttttgagaaaacaaggaggttctcgaactagaatacaaaggaaaag	98
				Poaceae_5	0.33	atccgtgttttgagaaaacaaggggttctcgaactagaatacaaaggaaaag	23
	Magnoliopsida	Apiales	Apiaceae	Apiaceae_3	0.33	atcctattttccaaaaacaaacaaaggcccagaaggtgaaaaaag	15
		Caryophyllales	Plumbaginaceae	*Limonium* _3	0.67	ctcccgctttccaaatcaaaaaag	242
			Amaranthaceae	*Chenopodium murale*	0.33	ctccttttgtcaaaagcaaaaaatactcaaaagaaaaaaataataaaaaagcaagaaaaaaaaag	81
				Chenopodium_1	0.33	ctcctttttcaaaagcaagaataaaaaaaaag	10
				Patellifolia_1	0.67	ctcctttttttttcaaaagcaaaaaaagaaggattcagaaaacaagaataaaaaaag	56
				Patellifolia_4	0.33	ctcctttttttttcaaaagcaaaaaaagaaggattcagaaaacaagaataaaaaag	11
		Fabales	Fabaceae	Fabaceae_4	0.33	atcctgttttccgaaaacaaagaaaagttcataaagtgataataaaaaagg	105
				Fabaceae_7	0.33	atcctgttttctgaaaacaaagaaaaattaagaaagttataataaaaaagg	46
		Lamiales	Plantaginaceae	*Plantago major*	0.33	atcctgtcttctcaaaataaagttcagaaagcgaaaaggg	19
		Solanales	Convolvulaceae	Ipomoea_1	0.33	atcctgttttccgaaaacaaacaaaagttcagaaaaaaag	18
Arthropoda	Arachnida	Araneae	Salticidae	*Stenaelurillus nigricaudus*	1.00	ttttttacaagtcgaacagacttcctttattaaatcttgctttaattaggttattaattcaacatcgaggtcacaatctatttttaatataaggactttgaaaaataa	1434
	Insecta	Coleoptera	Cerambycidae	Cerambycidae_1	0.33	ggtttcaaaggtcgaacagactcaataataaagctactacacctaaaattaaccttaatccaacatcgaggtcgcaaacttcactttcgatttgaactctctagcaaaa	105
			Elateridae	Elateridae_1	0.33	ggtttcaaaagtcgaacagactcaataataaagctaccatacctaaaattaaccttaatccaacatcgaggtcgcaaacttcactttcgatttgaactctctagcaaga	132
		Diptera	NI	Diptera_3	1.00	gaaataaaagtcgaacagacttaaagcttaaacgactacacctaagctttatcttaatccaacatcgaggtcgcaatcctttttatcgatatggactctccaaaaaga	4173
				Diptera_9	1.00	gaatttaaaagtcgaacagacttaacctttaagcggctacacctaaaaatatctcttaatccaacatcgaggtcgcaatcttttttatcgatatgaactctcaaaaaaa	2729
			Phoridae	*Megaselia* _1	0.67	gaatttaaaagtcgaacagactttaaatttaagcttctgcacctaaaattatatcttaatccaacatcgaggtcgcaatcatttttatcaataagaactctccaaaaata	875
		Hemiptera	Aphididae	*Aphis* _1	0.67	gaatttaaaggtcgaacagacctaatatttaaaattttgcacctaaaattaatcttaattcaacatcgaggtcgcaaactaatttttaaatttgaacttaaaaaattaa	213
			Cicadellidae	*Orosius* _1	1.00	gaattcaagagtcgaacagactcaccttataagggggctgccccttaaagttttcttaattcaacatcgaggtcgcaaaagaaagtatagatgtgaactccccactccaa	17894
			Miridae	*Campylomma* _1	0.67	aaatttaaaggtcgaacagacctagtattcaatcttatactcctgaaacttattttaatccaacatcgaggtcgcaaactattttatcgataagaactctcaaaaataa	362
		Hymenoptera	Apidae	*Bombus* _1	1.00	gatttcaaaagtcgaacagacttaatatttaaactgctgcatttaatatttatcttaattcaacatcgaggtcgcaatcttctttgttaatatggtctttaaaaagaaa	803
			Formicidae	Formicidae_1	0.67	gattttaaaagtcgaacagacttaaattttaatagtaattaaattttatcttaattcaacatcgaggtcgcaaacattttattaaataaatctttgataaaata	310
				Camponotus _2	1.00	aattttaatagtcgaacagactaaataattaaacttctccatttaatcttaattttaattcaacatcgaggtcgcaatcatctttataaataagatctctataaaaata	1250
			Thynnidae	Thynnidae_1	0.33	gaatttaatagtcgaacagactaaatctattagactcctgcatctaatttttatcttaattcaacatcgaggtcacaatcatttttatagatttggtctctaaaaaaata	111
		Lepidoptera	NI	Lepidoptera_29	0.67	gattttaatgatcgaacagatcaaaattttaaacttttgcatttaaattttatcttaatccaacatcgaggtcgcaaactttttttttatttgaactaaaaaaaaaaa	265
		Orthoptera	Gryllidae	Gryllidae_1	1.00	gaatttaatggtcgaacagaccaattaattaaacttctgcacctaatcatattcttaatccaacatcgaggtcgcaatcttcattatcaataagaactctccaataaaa	647
		Zygentoma	Lepismatidae	*Thermobia domestica*	0.67	gaatttaaaggtcgaacagaccttttttcctaagcttctgcacctaagaaacatcttaattcaacatcgaggtcgcaatccttcttattgataaggactcgtaaagaaga	260
	Malacostraca	Decapoda	NI	Decapoda_1	1.00	gattttaatagtcgaacagactatccccttaaaaaactgcccctaaaagaaatcttaattcaacatcgaggtcgcaaccattaatgtagatatgaactcttaaacaata	1387
