# Supplementary material for: Digging in a 120 years-old lunch: What can we learn from collection specimens of extinct species?
Source: PLoS One. 2022 Jul 6;17(7):e0270032. doi: 10.1371/journal.pone.0270032 (PMC9258829; doi:10.1371/journal.pone.0270032)
Supplement: S1 Appendix — (PDF) [file pone.0270032.s004.pdf]

Abstract in Portuguese

Estudar espécimes de colecções é frequentemente a única maneira de desvendar informações sobre extinções recentes. Estes podem revelar conhecimento sobre ameaças e características das espécies relacionados com a extinção e contribuir, por extrapolação, para a conservação das espécies existentes. No entanto, raramente foram usados métodos moleculares de sequenciação de alto rendimento para desvendar informação sobre a ecologia das mesmas. As espécies insulares são especialmente propensas à extinção. Estudámos os conteúdos do intestino de três espécimes de lagarto gigante das ilhas de Cabo Verde *Chioninia coctei* extinto usando microscopia e *metabarcoding* de ADN. A presença de nemátodes *Tachygonetria* sugere que as plantas eram importantes itens alimentares. A nossa abordagem de *metabarcoding* também identificou plantas e, adicionalmente, invertebrados, apoiando a hipótese de uma dieta generalista do *C. coctei*. A ausência de vertebrados nos conteúdos digestivos pode indicar o declínio das aves marinhas nas ilhas Desertas que pode ter contribuído para a debilitação do lagarto gigante, já infligido pela perseguição e secas intensas. Este estudo contribui para lançar luz sobre os papéis tróficos desta espécie extinta enigmática e enfatiza a necessidade de desenvolver planos de conservação holísticos para espécies insulares ameaçadas. Adicionalmente, ilustra o potencial de integrar métodos moleculares recentes com abordagens tradicionais para estudar espécimes de museus para ajudar a resolver puzzles ecológicos noutros ecossistemas.
